# Assessment of Nursing Students’ Awareness toward Ionizing Radiation: Cross-Sectional Study

**DOI:** 10.3390/nursrep13020075

**Published:** 2023-06-05

**Authors:** Suliman Salih, Mohd Nazmi Nordin, Ajnas Alkatheeri, Alanoud Nasser, Mezna Saif, Zuwaina Abdallah, Aljazi Alderei, Laila Ali Faisal, Mustafa Alhasan, Mohamed Hasaneen

**Affiliations:** 1 Department of Radiography and Medical Imaging, Fatima College of Health Sciences, Abu Dhabi 3798, United Arab Emirates; mohd.nordin@fchs.ac.ae (M.N.N.); alanoodnasser_1999@outlook.com (A.N.); almazrouie45@gmail.com (M.S.); zwina-121@hotmail.com (Z.A.); aljazi.alderei@outlook.com (A.A.); la.bk.7026@gmail.com (L.A.F.); mohamed.hasaneen@fchs.ac.ae (M.H.); 2 National Cancer Institute, University of Gezira, Wad Madani 2667, Sudan; 3 Faculty of Applied Medical Sciences, Department of Allied Medical Sciences, Jordan University of Science and Technology, Irbid 22110, Jordan; mkalhasan@just.edu.jo

**Keywords:** radiation protection, nursing students, ionizing radiation hazards

## Abstract

Among healthcare workers, nurses are considered the core of healthcare auth–info services in healthcare facilities because of their responsibilities and duties toward patients. All healthcare professionals, especially nurses, must be completely knowledgeable about the hazards of ionizing radiation, and the most effective radiation protection techniques. This study assessed the attitude and awareness toward radiation protection among final-year nursing students in the Fatima College of Health Sciences (FCHS) campuses. An online cross-sectional survey was conducted between March and April 2022. A total of 200 out of 224 female participants ranging from 18 to 30 years old agreed to participate in the study. The highest percentage of final-year nursing students did not attend any radiation protection course (52%). The results of the last section of the survey indicate a lack of awareness of basic radiation protection knowledge among final-year nursing students in FCHS campuses (less than 80%). The results showed a lack of knowledge and poor attitude toward radiation hazards and radiation protection from final-year nursing students in the FCHS. Formal education about basic radiation and radiation is recommended in the nursing program for safe clinical practice.

## 1. Introduction

Ionizing radiation is a term that describes the type of energy that can remove an electron from atoms and molecules of materials such as air, water, and living tissue [1]. Since its inception, the use of ionizing radiation in medical practice has evolved. Regarding convenience and diagnostic and therapeutic effectiveness, they provide significant benefits to patients. According to Tavakoli, SeilanianToosi, and Saadatjou (2003), it has been estimated that about 30–50% of critical medical decisions are based on X-ray examinations. Furthermore, clinicians who use X-rays as diagnostic tools must have a thorough understanding and accurate radiation protection knowledge concerning the benefits and drawbacks of the procedure [2,3]. This is because occupational radiation protection involves procedures and protocols that ensure the safety of patients and medical staff who are involved in imaging.

Moreover, health facilities should provide workers with radiation protection tools such as lead gowns or aprons. This is applicable to those who are most likely to be involved in any ionizing radiation procedures, such as performing mobile X-ray examinations in the ward. Using radiation protection and implementing radiation protection protocols while scanning patients can minimize the hazards of ionizing radiation. Organizations and their management are committed to radiological protection in their departments. All parties involved are required to contribute to the reduction in and control of exposure and to provide adequate and relevant training programs. Increased productivity and retention of expert healthcare staff in healthcare facilities that have radiation protection guidelines have increased radiation risk awareness, reduced unsafe practices, and improved the quality of their radiation protection programs.

The increased use of ionization radiation raises concerns about radiation protection for both healthcare staff and patients. The Radiation Protection Society states that safety standards and procedures should be implemented to reduce the hazards of radiation exposure among patients and healthcare staff. Similarly, the Code of Federal Regulations established the ‘As Low as Reasonably Achievable’ (ALARA) principle to ensure that all methods decrease radiation exposure while using radiation during imaging patients [4]. Health hazards can occur to patients and medical staff who are exposed to or work with ionizing radiation such as skin burns, hair loss, and acute radiation syndrome. The majority of these radiation effects require very large amounts of radiation, which would not occur from general X-rays but could (very rarely) occur from computed tomography (CT) or interventional radiology procedures. In addition, long-term exposure to ionizing radiation for a long period increases the risk of cancer [2,5,6].

After graduation, many nurses work in the radiology or diagnostic imaging departments. In the United Arab Emirates (UAE), almost all dental clinics with X-ray equipment such as orthopantomography machines are operated by nurses. Heidari et al. (2017) stated that nurses are at the core of healthcare services. The study discusses the vital role of nurses in the healthcare services that are needed by healthcare facilities to understand the significance of providing a safe work environment for their staff, increasing their productivity, and ensuring their safety [7]. In rare situations, nurses escort patients from wards to the radiology department and have a role in performing the radiation procedures that lead them to be exposed to scatter radiation. When dealing with unstable critically ill patients who require a high level of technologic monitoring and physiologic support, patients may remain stable or experience a subtle or abrupt deterioration in status, which, in some cases, may progress to cardiorespiratory collapse; this would require a nurse to assist and provide care and support to the patient during the examinations in the radiology department, which increases their chance of being exposed to scatter radiation. Hence, they are required to aware of, educated about, and abide by radiation safety guidelines to ensure their safety [8,9].

Although radiation awareness and radiation protection are important issues of safety for health practitioners involved in services using ionizing radiation, there are no published data from the UAE on this topic and there are no formal courses about radiation hazards and protection in the curriculum of nursing program at the Fatima College of Health Sciences (FCHS). Identifying gaps in knowledge in this field can help improve the nursing curriculum in academic institutions. Therefore, the primary objective of this study was to assess the attitudes and awareness of final-year nursing students at the FCHS toward ionizing radiation and radiation protection.

## 2. Materials and Methods

### 2.1. Study Design and Study Population

A prospective cross-sectional questionnaire survey was conducted to collected data from FCHS final-year nursing students on all the FCHS campuses (Abu Dhabi, Al Ain, Ajman, and Al Dhafra), which is the only governmental health science higher education institute in the UAE for women. The STROBE reporting guidelines were followed in reporting the data [10]. The authors adopted a validated survey used by Salih et al. [11]. The questions of the questionnaire had been checked to make sure that they are clear and understandable.

In 2021–2022, around 224 nursing students were in their final year in all the FCHS campuses. Based on the sample size calculator (the Epi-Info version 7 StatCalc from the Center for Disease Control and Prevention (CDC)), the sufficient amount of data to generalize this research outcome on FCHS’s whole nurse student population is N = 99 (the inadequate knowledge rate is 50%, with an 80% confidence interval and a 5% margin error).

To reduce the bias, random sampling was used, so the entire target population has an equal opportunity of being chosen. All the survey answers were treated anonymously and confidentially, so the participants were more willing to answer honestly.

### 2.2. Method of the Survey

An online questionnaire was developed and sent via e-mail to 224 final-year nursing students between March and April 2022 from various FCHS campuses (Al Ain, Abu Dhabi, Ajman, and Al Dhafra) across the UAE. This sample was considered to have the highest number of nursing students among all nursing programs for all higher education institutes in the UAE. The questionnaire contained 4 components. In the first of the consent forms, the background and aims of the research are explained to the participants in order to acquire their consent to continue and complete the questionnaire. The second component contained demographic information about the individuals. The third component consisted of fundamental questions that assessed the nurses’ knowledge and attitudes regarding ionizing radiation and radiation protection. This part included 3 multiple-choice questions (MCQs) and 2 checkboxes. The final component consisted of 15 MCQs assessing the nurses’ knowledge and attitudes regarding radiation hazards and safety.

### 2.3. Data Collection and Analysis

Random nursing final-year students from the FCHS were invited to participate in the study, and 200 of them accepted the invitation. The data were analyzed using SAS software version 9.4 (SAS Institute, Inc., Cary, NC, USA), and a *p*-value less than 0.05 was considered as significant. The demographic data were evaluated with descriptive statistics and summarized as percentages, while the data of the radiation knowledge were summarized as percentages, standard deviation, and mean. All the responses from the participants were handled anonymously and in accordance with the established guidelines for a good research approach.

### 2.4. Ethical Consideration

Ethical approval was obtained from the Fatima College Research Ethics Committee (no. INTSTF016RMI20). The participants’ agreement was obtained through a survey consent form. The data that support the findings of this study are available from the corresponding author, [Suliman Salih], upon reasonable request.

## 3. Results

### 3.1. General Information of Participants

A total of 200 participants from various FCHS campuses completed the survey. The highest response percentage was from the Al Ain campus (33%), and the lowest feedback came from the Al Dhafra campus (16%). Most participants’ ages ranged from 21–23 years old (64%), and only 2.5% of participants’ ages were 24 years or older (Table 1).

### 3.2. Nurses’ Attitude toward Ionising Radiation and Radiation Protection

Table 2 shows a summary of the nurses’ attitudes toward ionizing radiation and radiation protection. Most students did not attend radiation protection courses (52%, Mean = 0.48, Standard Deviation = 0.501), and the majority (71.86%, Mean = 0. 72, Standard Deviation = 0.5) preferred online radiation education courses. In addition, (77%, Mean = 2.6, Standard Deviation = 0.8) agreed that nurses should have basic knowledge of radiation protection, 17% disagreed, and six were not certain about the importance of having basic knowledge concerning radiation protection (6%). A total of 93% of final-year nursing students had been trained in the pediatric intensive care unit, 18% in angiography, and 8% in nuclear imaging facilities. During their clinical placements, (25%, Mean = 2.12, Standard Deviation = 1.30) of nursing students were exposed to scattered radiation once or twice a month during mobile X-ray examinations.

The correlation of demographic data and nurses’ attitudes toward ionizing radiation and radiation protection is analyzed. In this research, the statistical significance was set at *p* < 0.05, implying a significant correlation between those demographic data and radiation attitude toward radiation. The Pearson Chi-square test and cross-tabulation analysis showed that attending radiation protection courses, being trained in medical imaging facilities, and being exposed to radiation during clinical training correlate with the student campus. Nursing students from the Al Ain campus recorded significantly higher levels of radiation education and training and were frequently exposed to scatter radiation compared to the students from other FCHS campuses (*p* = 0.001) (Table 3).

### 3.3. Nursing Students’ Awareness on Radiation Protection and Hazard

Overall, Table 4 demonstrates the awareness of radiation protection and hazards among 200 final-year nursing students from the different FCHS campuses. This section includes the results of 15 questions that were used to assess their awareness toward radiation protection and hazards.

By referring to the table below, our findings showed that most students (72%, mean = 1.3, standard deviation = 0.91) chose the correct answer for the abbreviation of the term ALARA but not how to apply it in clinical practice (51%, mean = 1.23, standard deviation = 0.85). Moreover, questions from eight to ten aimed to assess the student’s ability to differentiate between ionizing and non-ionizing radiation (66.33%, mean = 1.5, standard deviation = 0.7) and (76.38%, mean = 1.99, standard deviation = 0.49) and (70%, mean = 1.6, standard deviation = 0.73) of the total respondents, respectively. Multiple linear regression was used to test if attending a radiation protection course significantly predicted nursing students’ awareness on radiation. The nursing students who have attended a course on radiation education and protection did not record higher levels of this knowledge area than the nurses who had not attended any course (R^2^ = 0.012, F (3,195) = 0.774, *p* = 0.142).

The results showed that the participants had good knowledge regarding the radiation hazard on normal tissue and its effect on the fetus and pregnancy. The majority were able to recognize that frequent exposure to higher ionizing radiation is harmful (73.50%, mean = 1.4, standard deviation = 0.69). Additionally, (76.38%, mean = 1.7, standard deviation = 0.63), it is known that radiation poses a hazard to the pregnancy. The nursing students chose lead as the best material with radiation shielding capabilities (72%, mean = 1.9, standard deviation = 1.52). The result showed a statistical correlation between nurses having trained in imaging facilities and having better knowledge regarding radiation safety and protection, (*p* value= 0.050).

In addition, around 50% of the students had good knowledge regarding the annual occupational radiation dose limit for workers and pregnant workers and the effectiveness of X-ray examination on the dose. Only 39.70%, mean = 1.9, standard deviation = 0.82) chose the correct dose limit for public exposure. The Pearson Chi-Square test presented a correlation between the student campus and their knowledge about the radiation dose (*p* < 0.001).

## 4. Discussion

The purpose of this study was to assess the attitudes and awareness of final-year nursing students toward basic radiation hazards and protection in all four FCHS campuses. Working in hospitals can expose nurses to unnecessary radiation. Keeping in mind that final-year nursing students spend most of their training time in hospitals and will become future nursing practitioners. Thus, a basic knowledge of radiation protection is expected to be gained. For this study, 80% was the minimum accepted limit for the success rate because the targeted students were final-year students. In this study, 200 of 224 participants responded to the survey. Most responses were from the Al Ain campus (33%), and the lowest response was from the Al Dhafra (16%) campus. Most participants’ ages ranged from 21–23 years old (64%), and only (2.50%) of participants’ ages ranged from 24 years and above (Table 1).

**Figure 1:**
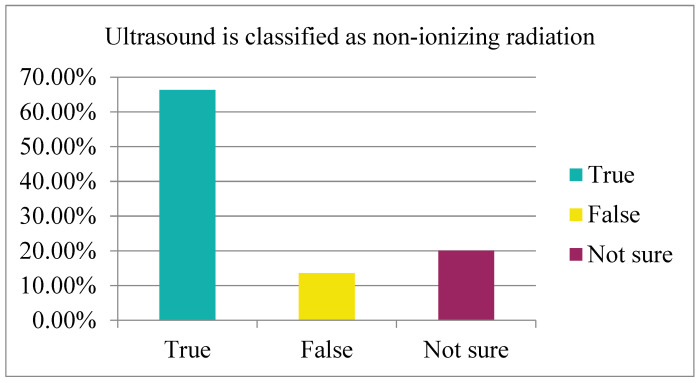
The final-year nursing students’ knowledge about ultrasound radiation type. Most participants chose the correct answer (66.33%).

The results showed that 77% of the participants agreed that nursing students should be provided with basic knowledge, as in Figure 1. This finding agrees with a study in 2015 by Alotaibi et al. [12]. They discussed the essence of enhancing radiation programs in nursing education to improve the safety of patients and nurses. The study concluded that there were inadequate radiation principles among nurses that threaten patients’ safety and suggested improving nurses’ radiation knowledge to improve the healthcare of patients [12]. A similar study conducted by Essien and Nyong (2016) found that most nurses were not very confident in their knowledge of ionizing radiation and its consequences owing to the lack of formal training; however, they accepted the importance of radiation protection and the importance of radiation in diagnostic radiology [13]. Moreover, this result coincides with the radiation protection recommendations of the international radiation protection guidelines, which stated that the major principles and practice policies must be developed and used to educate healthcare workers to minimize radiation risks [14,15].

The findings showed that (48%) of the participants attended radiation protection courses and (52%) did not attend any radiation protection courses. This could lead to a low level of awareness. This finding is concurrent with a study conducted by Rafique et al. (2021), which showed that nurses have the lowest awareness of radiation protection among the medical staff because of the underestimation of attending radiation safety awareness courses [16,17]. Moreover, Dianati et al. (2014) assessed nurses’ understanding of radiation protection as direct caregivers [18]. The results suggest that 90.0% of the respondents were unaware of the Protection of Persons Undergoing Medical Exposure or Treatment (POPUMET) legislation and that radiation protection training was poorly attended. The study recommends that deliberate efforts be made at the researched healthcare institutions to train nurses on the POPUMET requirements [18]. According to our findings, the number of final-year nursing students that have been exposed to radiation during clinical training at least once per month reached 25%, twice per month at 25%, three times per month at 19.50%, four times per month at 18.50%, and more than five times at about 1%. This agrees with the findings of another study, which reported that the use of radiation in medical facilities continues to increase with time. It is important to continue to minimize the radiation exposure of medical workers [13,19].

Misinformation regarding radiation and fear of radiation hazards are issues even among healthcare providers. Our results showed in Question 12 (Table 4) that the highest percentage of participants (47%) chose cancer, which is the right answer and is considered a potential factor of fear of cancer among nurses. Moreover, for Question 13, a total response of 73.50% believed that frequent exposure to higher ionizing radiation is harmful, which is the correct answer. This result is supported by the study by Dhingra et al. (2017), who found that nursing students had a perceived fear of radiation exposure. The results showed that nursing students had anxiety about exposure to radiation and the link between cancer and infertility [20,21].

The potential harm caused by radiation during fluoroscopy can be significant for medical professionals, particularly nurses. No one should be placed in the room during the CT scan. Staff members are more likely to be in the room for interventional procedures. Therefore, health practitioners who may be involved in radiology procedures should be aware of the ALARA principles [14,15]. Our findings show that most students choose the correct answer for the abbreviation of the term ALARA but not how to apply it during clinical practice. As shown in Question 16 (Table 4), 49% of participants chose the right action and application of the ALARA principle on what the nurses should do during imaging scans if the nurse cannot leave the examination room; 11% chose the answer ‘do nothing’ and 40% chose the wrong answer. The results showed a lack in final-year nursing students’ awareness of basic radiation protection since ALARA is considered one of the important basic principles of radiation. Similarly, a study evaluated the knowledge and practice of medical professionals using a questionnaire and showed that only 47.7% of participants could comprehend the concept of ALARA [22]. A part of this study survey, which covered questions concerning the ALARA principle, showed an interesting point: even though most participants answered right, they did not pass the success rate. Further, 72% of the students could identify the meaning of ALARA, while 28% chose the wrong answer. Another question concerning ALARA was about the rules that it stands for which gained a much lower percentage: 27.50% of students chose the right answer while 72.50% of the students chose the wrong answers. The results for Question 14 show that 26.5% of the nursing students think that increasing their distance from the source of radiation will increase the dose, 16% of the students think the dose will remain the same, and only 57% chose the correct answer which is ‘decrease’. This confirmed the nursing students’ understanding of how to apply the ALARA principle. Depending on the provided statistics, most students figured out the meaning of ALARA but not how to apply ALARA principles during their clinical practice [20].

Participating in final-year nursing students’ knowledge of sources of ionizing radiation scored a high percentage. The majority (66.33%) believed that ultrasound is classified as non-ionizing radiation, 13.57% wrongly believed that it is ionizing radiation, and 20.10% were not sure. As for CT modality, most (70%) nurses said that CT scanning is classified as ionizing radiation, 14.50% wrongly answered that CT is considered as non-ionizing, and 15.50% were not sure. As for the X-ray, most students (76%) answered it correctly; however, some (12.5%) believed that it was a form of non-ionizing radiation, while others (11.5%) were not sure about it. The results showed there is a lack of awareness regarding the source of ionizing and non-ionizing radiation since none of the three questions reached the (80%) limit for the right answer. Yurt (2014) reported that health professionals have limited knowledge about ionizing radiation [22]. The study used a questionnaire to evaluate the knowledge level of ionizing radiation and the dose limit for occupational exposure. The participants were asked to complete 42 questions regarding the safe dose of ionizing radiation. Similarly, the results showed that knowledge levels were very limited among health professionals using ionizing radiation in their work. According to our findings, the students showed a lack of awareness regarding the dose limit for occupational exposure (workers). Only 46.73% answered correctly, whereas 30.65% were unsure. This was similar to Yurt’s (2014) finding [20]. In another question regarding the dose limit for occupational exposure (pregnant worker), only 44.50% chose the correct answer and 55.50% chose the wrong answer or were not sure. One question was about the dose limit for public exposure, which only 39.70% of the total respondents answered correctly, while the majority chose the wrong answer (31.66%) and 28.64% were unsure. All the questions regarding the radiation dose limit did not reach 80% for the correct answer, indicating a lack of awareness among final-year nursing students, similar to Yurt’s result [20].

Healthcare facility management must create safety guidelines in the radiology department to ensure the safety of patients and medical staff. In addition, management needs to provide nurses with personal protection equipment in the radiology department [23]. Assistant nurses represent a higher rate of exposure to radiation hazards than licensed nurses and registered and advanced practice registered nurses. Babaloui et al. (2018) claimed that work experience correlates with radiation awareness and that nurses with higher work experience reflect more knowledge of radiation hazards. Contrastingly, the study was conducted with nurses from different wards that reported low radiation awareness that threatens both nurses’ and patients’ safety [24,25]. Therefore, educating health practitioners on radiation best practices is a crucial step in optimizing safe radiation practices [4].

Rahimi et al. (2021) conducted a cross-sectional questionnaire survey of Malaysian nurses. The current results showed a high level of awareness regarding radiation protection and safety [9]. This agrees with our results. This can be explained by the fact that Malaysian nurses’ colleges might have introduced basic radiation and radiation protection in their curricula. This explanation is confirmed by other studies, which reported that education on radiation protection topics is a significant factor in the orientation and implementation of radiation protection principles in clinical practice and in reducing exposure to radiation hazards [11,21,26].

## 5. Limitations

The objective of this study was to identify the attitudes and awareness toward radiation hazards and protection awareness among final-year nursing students at the FCHS campuses. However, because the study was limited to FCHS nursing students, the results cannot be generalized to all nursing students in the UAE. By the way, these results can be used as basic data to support the establishment of policies to improve radiation protection environments for ER nurses in the future.

Additionally, there is no monitoring method to determine whether the participants will respond to the cognitive questions using their expertise or whether they will consult another resource, such as Google. Additionally, the questionnaire for nursing students did not include questions about radiation risks associated with radiological examinations for patients and staff. On the other hand, the question about the value of dose limits could have been omitted.

## 6. Conclusions

This study shows the lack of basic and specialized radiation protection and safety owing to the absence of formal courses on basic radiation hazards and radiation protection in their curriculum. Formal education about basic radiation and radiation is recommended in the nursing program of the FCHS for safe clinical practice.

## Figures and Tables

**Table 1:** Demographic data.

Item	Percentage
Student campus	Al Dhafra = 16% Abu Dhabi = 26% Ajman = 25% Al Ain = 33%
Age group (years)	18–20 = 33% 21–23 = 64.50% ≥24 = 2.50%

**Table 2:** Radiation protection education and training received by nursing students.

Questions	Response (%)
1. Have you attended any radiation protection courses?	Yes = 48.00% No = 52.00%
2. Personally, which method of education do you think will help?	Online courses = 71.86% Formal course at college = 63.82% Training = 69.35% Workshop = 55.28% Other (please specify) = 0.00%
3. In your opinion, is it important to provide nurses with the basic knowledge concerning radiation protection?	Agree = 77.00% Disagree = 17.00% Not sure = 6.00%
4. Have you trained in any of these medical wards or imaging facilities?	Angiography = 18.00% Nuclear medicine = 8.00% Pediatric intensive care unit = 93.00% Other (please specify) = 11.50%
5. How frequently have you been exposed to radiation during your clinical placement/training during mobile X-ray examination per month?	None = 11.00% Once = 25.00% Twice = 25.00% Three-time = 19.50% Four times = 18.50% More than five times = 1.00%

**Table 3:** Student campus correlated with nurses’ attitudes toward ionizing radiation and radiation protection.

Pearson Chi-Square Tests		
		Student Campus
Nurses’ attitudes toward ionizing radiation and radiation protection	Chi-square	66.959
	df	15
	Sig.	<0.001 *
Results are based on nonempty rows and columns in each innermost subtable.		

* The Chi-square statistic is significant at the 0.05 level.

**Table 4:** Nursing students’ awareness on radiation protection and hazards.

Questions	Response (%)
6. ALARA is an acronym of…	As Low as Reasonably Acceptable = 19.50% As Low as Reasonably Achievable = 72.00% As Low as Reasonably Acquirable = 8.50%
7. ALARA rules of radiation protection are time, distance, shielding, and collimation.	True = 51.00% False = 27.50% Not sure = 21.50%
8. Ultrasound is classified as non-ionizing radiation.	True = 66.33% False = 13.57% Not sure = 20.10%
9. X-rays are a…	Form of non-ionizing radiation = 12.5% Form of ionizing radiation = 76% Not sure = 11.5%
10. Computed tomography is classified as ionizing radiation.	True = 70.00% False = 14.50% Not sure = 15.50%
11. Ionizing radiation can affect pregnant women.	True = 76.38% False = 9.05% Not sure = 14.57%
12. All the following are considered deterministic effects of radiation EXCEPT?	Diminished sperm out = 22.50% Epilation = 15.50% Cancer = 47.00% Skin erythema = 15.00%
13. Frequent exposure to higher ionizing radiation examination is?	Harmful = 73.50% Beneficial = 14.50% Not sure = 12.00%
14. What material is characterized as having the best radiation shielding capabilities?	Aluminum = 18.5% Lead = 72% Iron = 9%
15. What is the dose limit for occupational exposure (pregnant worker)?	1 mSv/year = 44.50% 10 mSv/year = 27.00% Not sure = 28.50%
16. As a nurse increases his/her distance from the source of radiation, the dose will…	Increase = 26.50% Decrease = 57.50% Remain the same = 16.00%
17. What is the dose limit for occupational exposure (worker)?	20 mSv/year = 46.73% 50 mSv/year = 22.61% Not sure = 30.65%
18. When the nurses cannot leave the room during an X-ray exam, which of these techniques will reduce their occupational dose?	Stepping away as possible from the X-ray source = 21% Wearing a lead apron = 19% Do nothing = 11% Both 1&2 = 49%
19. What is the dose limit for public exposure?	1 mSv/year = 39.70% 20 mSv/year = 31.66% Not sure = 28.64%

## Data Availability

The data that support the findings of this study are available from the corresponding author, [Suliman Salih], upon reasonable request.
